# Supplementary material for: Lin−CCR2+ hematopoietic stem and progenitor cells overcome resistance to PD-1 blockade
Source: Nat Commun. 2018 Oct 17;9:4313. doi: 10.1038/s41467-018-06182-5 (PMC6192988; doi:10.1038/s41467-018-06182-5)
Supplement: Supplementary file 1 — Supplementary Information [file 41467_2018_6182_MOESM1_ESM.pdf]

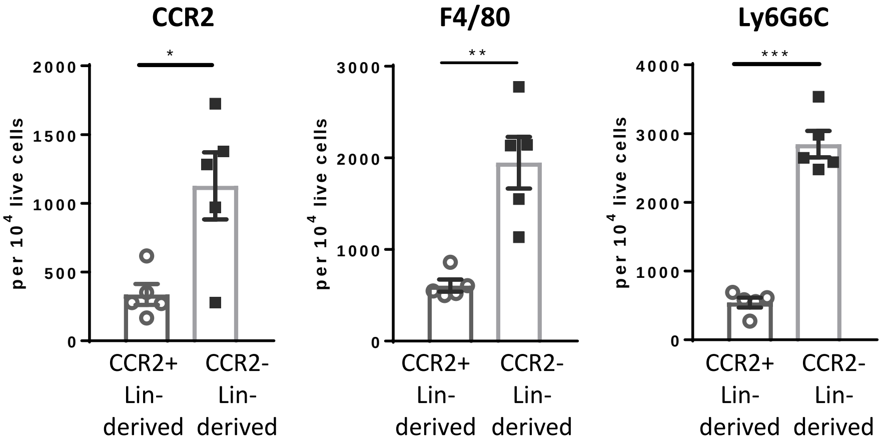

SUPPLEMENTARY FIGURE 1. CCR2-Lin- derived cells upregulate MDCS markers. KR158B glioma tumor bearing mice received adoptive transfer of equal numbers of GFP+CCR2-HSCs and DsRed+CCR2+HSCs. Tumors were excised and GFP+ and DsRed+ cells phenotyped for markers of MDSCs. (*p=0.003, **p=0.0138, ***p=0.0001, unpaired t-test, n=5/group). All error bars represent s.e.m.

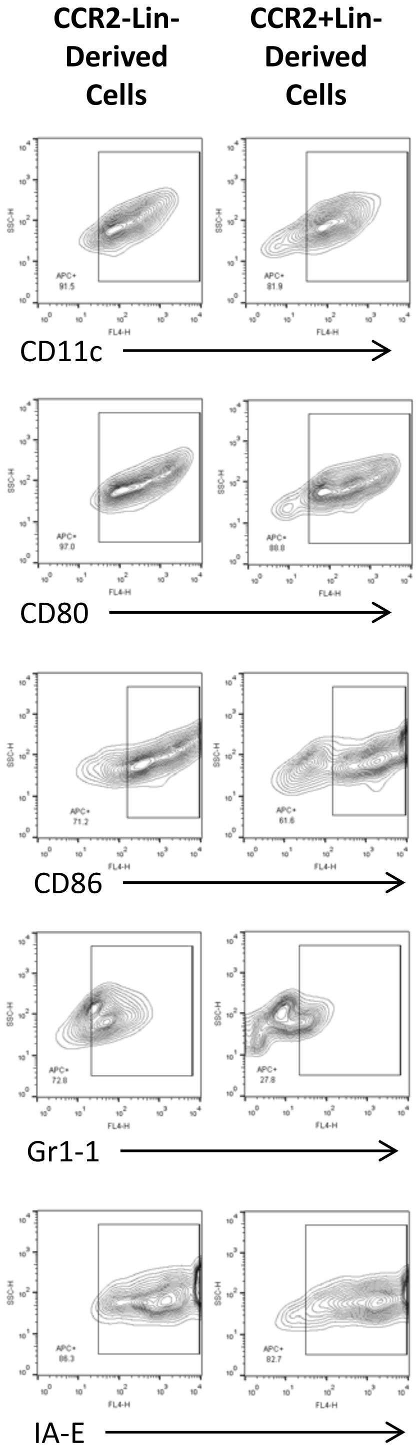

SUPPLEMENTARY FIGURE 2. CCR2+HSCs upregulate dendritic cell phenotype. CCR2-HSCs and CCR2+HSCs were isolated from bone marrow and placed in dendritic cell culture conditions for nine days and phenotyped.
